# Candidate genes for migration do not distinguish migratory and non-migratory birds

**DOI:** 10.1007/s00359-017-1184-6

**Published:** 2017-06-05

**Authors:** Juan S. Lugo Ramos, Kira E. Delmore, Miriam Liedvogel

**Affiliations:** Max Planck Institute for Evolutionary Biology, AG Behavioural Genomics, August-Thienemann-Str. 2, 24306 Plön, Germany

**Keywords:** Candidate genes, Migratory traits, Bird migration, Next-generation sequencing, Genomic data

## Abstract

Migratory traits in birds have been shown to have a strong heritable component and several candidate genes have been suggested to control these migratory traits. To investigate if the genetic makeup of one or a set of these candidate genes can be used to identify a general pattern between migratory and non-migratory birds, we extracted genomic sequence data for 25 hypothesised candidate genes for migration from 70 available genomes across all orders of Aves and characterised sequence divergence between migratory and non-migratory phenotypes. When examining each gene separately across all species, we did not identify any genetic variants in candidate genes that distinguished migrants from non-migrants; any resulting pattern was driven by the phylogenetic signal. This was true for each gene analysed independently, but also for concatenated sequence alignments of all candidate genes combined. We also attempted to distinguish between migrant and non-migrants using structural features at four candidate genes that have previously been reported to show associated with migratory behaviour but did not pick up a signal for migratory phenotype here either. Finally, a screen for d*N*/d*S* ratio across all focal candidate genes to probe for putative features of selection did not uncover a pattern, though this might not be expected given the broad phylogenetic scale used here. Our study demonstrates the potential of public genomic data to test for general patterns of migratory gene candidates in a cross-species comparative context, and raise questions on the applicability of candidate gene approaches in a macro-evolutionary context to understand the genetic architecture of migratory behaviour.

**Electronic supplementary material:**

The online version of this article (doi:10.1007/s00359-017-1184-6) contains supplementary material, which is available to authorized users.

## Introduction

Bird migration is one of the most fascinating and well-studied behaviours among birds, including work on the physiological and morphological adaptations required for successful migration and ecological correlates of this behaviour. Considerable interest has also focused on understanding how variation in the migratory phenotype is generated, with several studies demonstrating high variability for various migratory traits using selective breeding studies (e.g. Berthold et al. 1992), displacement experiments (e.g. Perdeck 1958; Chernetsov et al. 2008) and quantitative genetics analyses (e.g. Pulido and Berthold 2010). Nevertheless, the underlying genetic architecture shaping this behaviour remains poorly understood (Liedvogel et al. 2011; Delmore and Liedvogel 2016). One popular approach to enhance our understanding of the molecular basis of migratory traits has been the use of candidate genes for behavioural traits suggested to contribute to variation in migratory phenotype. These candidates are often selected by their molecularly characterised specific function in other (often model) organisms. A candidate gene approach attempts to identify an association between genetic variation in that gene and the focal phenotype, here our focal behaviour is migration. The underlying rationale of this approach is to focus on genetic variation in specific candidate regions of the genome that have been suggested to directly impact the function of the candidate gene and ultimately the target phenotype in other species as well. In the context of migration, the traits receiving most attention are circadian behaviour and personality traits (e.g. exploratory or anxiety-related behaviour); focal candidate genes include *CLOCK, ADCYAP1, CREB1* and *NPAS2* for circadian rhythm, and *DRD4* and *SERT* for personality traits (see Müller et al. 2011 as one of the pioneer studies in the context of migration).

*CLOCK* and *ADCYAP1* are among the most studied candidate genes in the field of bird migration. The overall pattern for *CLOCK* variability indicates a latitudinal cline in repeat lengths at the variable region, possibly reflecting local adaptation to seasonal variation at different latitudes. The circadian *CLOCK* gene is highly conserved among birds throughout most of its sequence with the exception of one C-terminal region that contains a variable poly-glutamine(Q) (poly-Q) motif with variability in the number of glutamine repeats both among and within species (e.g. Johnsen et al. 2007; Liedvogel et al. 2009; Bazzi et al. 2016; but also see Liedvogel and Sheldon 2010; Dor et al. 2011). This region has been suggested to influence the transcription activating potential of the protein, potentially altering rhythms in both physiology and behaviour. The aforementioned pattern of a latitudinal cline in lengths polymorphism has been recovered in several species and in a migration context has been suggested to reflect adaptive features related to migration. Similarly for *ADCYAP1,* a neuropeptide-coding gene, one 3′ UTR microsatellite length variation has shown associations with migratory behaviour in blackcaps (Müller et al. 2011; Mettler et al. 2015) with longer alleles associated with higher migratory activity. However, the pattern so far lacks consistency across other avian species (e.g. Peterson et al. 2013) in order to confirm its suggested role as regulatory unit of migratory behaviour.

More recently, Delmore et al. (2015a) used next-generation sequencing data to estimate genomic differentiation between two subspecies groups of *Catharus ustulatus* that exhibit differences in their migratory behaviour (both timing and orientation). They characterised the entire genome to probe for enrichment of candidate genes for migration in areas of elevated differentiation across the genome. The resulting list of candidates included 25 genes that had been identified using a literature search (Ruegg et al. 2014). As predicted, genes from the list of candidates were enriched in areas of elevated differentiation, suggesting selection around these candidates could not only contribute to variation documented in their migratory behaviour, but also help maintain differences between the groups. Delmore et al. (2016) expanded on this work using hybrids and a genome-wide association study to identify one region in particular that is associated with variation in migratory orientation and harbours the *CLOCK* gene.

Despite several clearly species-specific differences in behavioural traits that make up the migratory phenotype, all migratory birds have to meet a general consensus schedule of key adaptations in order to cope with the challenge of migration. Specifically, migratory birds need a set of navigation and orientation mechanisms to migrate successfully, and must complete several key physiological traits such as hyperphagia and feather moulting in time prior to their migratory journey. Migratory individuals also share common morphological and anatomical adaptations, such as elongated and more pointed wings, lighter bone structure, maybe also brain volume (e.g. Lockwood et al. 1998; Fuchs et al. 2014). Given this common set of features among migratory birds and demonstrations of both their heritability and potentially shared genetic basis (e.g. the aforementioned involvement of *CLOCK* and *ADCYAP1* across species), it has been postulated that there may be a shared set of genes for migration among birds (Berthold 1999).

Here, we test the hypothesis that sequence variation at one or a common set of suggested candidate genes for migration has been exploited to adapt to the requirements of migratory behaviour in different avian lineages using a cross-species comparative approach. Specifically, we focused on each of the 25 candidate genes for migration proposed by Ruegg et al. (2014) and compared patterns of genetic variability separately for each candidate gene between migratory and non-migratory species of birds. In addition to analyses per candidate gene, we also analysed concatenated sequence data from all candidate genes. This work benefited from the wealth of genomic data that have been accumulated in the last half-decade, with the assembly of several draft reference genomes for birds and large-scale initiative of the Avian phylogenomics project (http://avian.genomics.cn/en/index.html; more recently expanding to the B10K project). Our full dataset included 17 obligate migratory species, 32 sedentary or non-migratory species and 21 species with an additional intermediate movement phenotype (e.g. dispersive, partial migrant) (Table S1). We compare patterns of evolutionary divergence of each candidate gene using three different approaches: (1) comparing observed topologies for candidate genes to trees built using phylogenetic relationships with and without distinguishing migratory species from non-migratory species; (2) performing a gene-wide and branch-specific d*N*/d*S* analysis to identify if selective pressures on these candidate genes play a role related to migration; and (3) focussing on structural features that previous studies have shown to correlate with migratory behaviour (e.g. microsatellites at *ADCYAP1* and *CREB1* 3′ UTR regions and poly-Q regions in *NPAS2* and *CLOCK*), running linear models between these variants and two predictors of migratory behaviour. If genetic variation at candidate genes included in our study code for differences in migration, we predicted that gene trees based on candidate genes that play a role in shaping migratory behaviour would group migratory species together. Further, selective pressures on those candidate genes with a clear role in shaping the focal phenotype should be picked up in lineages with migratory species and linear models would show strong associations between genetic variation at structural features and predictors of migratory phenotype.

## Materials and methods

### Migratory phenotype characterisation

We classified each of the 70 species included in our study, according to their migratory phenotype. Our classification was based on a careful literature review of bird guides (Svensson et al. 1999), as well as BirdLife (http://birdlife.org) and Handbook of birds of the World (HBW) (http://www.hbw.com). We defined the following categories: clearly non-migratory (resident, sedentary) (0/R; *n* = 32), obligate migrant (2/M; *n* = 17). However, sometimes it is not easy to clearly define a species as either clearly non-migrant or obligate migrant, this is especially true when a migratory trait segregates within a population such as in partial migrants where only some individuals of the population migrate, consequently we added a third category (1; *n* = 21). This category includes partial migratory species (i.e. not all individuals of the population migrate), and species that exhibit other kind of migration-independent movement behaviour (e.g. dispersal, homing, foraging flights). For partial migrant species we used additional information of the individual used for generating the reference, such as date and geographical origin of sample collection, in order to clearly define migratory phenotype and grouped that individual/species accordingly whenever possible.

### Genome sequences, extraction and alignment

We downloaded genome sequences and annotations for most of the species used in our study from the NCBI database (Supplementary Table S1). We further included genome sequences of five additional migratory species here: Siberian stonechat *Saxicola maurus* (Van Doren et al. 2017), Swainson’s thrush *Catharus ustulatus* (Delmore et al. 2015a), European blackcap *Sylvia atricapilla* (Delmore et al., in preparation), Willow warbler *Phylloscopus trochilus* (Lundberg et al. 2017, accepted), and Greenish warbler *Phylloscopus trochiloides* (Irwin et al. 2016).

Once we had sequences data and annotation for all of the species, we used the Bedtools (Quinlan and Hall 2010) getfasta module to extract genomic sequences for each of the 25 candidate genes for every species. Sequences for unpublished genomes or genomes without annotations (for details see Table S1) were generated using Blastn and chicken cDNAs from the Ensembl database as a reference. All genomic sequences were aligned with MAFFT (Katoh and Standley 2013) and manually edited in AliView. Coding (CDS) sequences were also obtained from a multiple alignment of the genomic sequences and Ensemble cDNA sequences (including untranslated regions, UTRs) for the flycatcher, chicken, and Zebra Finch. Only sequences covering 50% or more of the chicken genes were considered for further analysis.

### Statistical analyses

#### Topological comparisons

Evolutionary trees were constructed for each candidate gene using whole genomic sequences and cDNA as reference, using a Neighbour Joining approach in MEGA v5.2 (Tamura et al. 2011). The reliability of the trees was evaluated performing a bootstrap analysis of 1000 replicates with the Kimura 2 Parameters model. To visualise the trees we used Figtree (http://tree.bio.ed.ac.uk/).

We compared the pattern of evolutionary divergence of these gene trees with three different hypothetical scenarios: divergence driven by phylogeny (‘phylogenetic topology’); divergence constrained by migration, i.e. different migratory phenotypes clustering in separate braches, while keeping the evolutionary relationship of the phylogenetic topology within each branch, (‘migratory phenotype topology’); and random divergence (‘random topology’). These comparisons were carried out for (a) the full dataset including all three migratory phenotypes; (b) a restricted dataset only contrasting exclusively obligatory migratory and completely non-migratory (resident) species, and (c) a clade-specific analysis exclusively focusing on the genus of Passeriformes, as this is the only monophyletic clade in our dataset with a sufficiently high number of species for both obligate migrants and non-migratory species, thus allowing for a more fine-tuned assessment on a narrower phylogenetic scale. The clade-specific subset allows us to test if the migratory phenotype might be controlled by a different clade-specific subset of genes. This comparative approach allows us to identify the presence or absence of general patterns, using genetic variation at candidate genes to distinguish between patterns related to phylogenetic relationships and migratory behaviour. The divergence driven by phylogeny (i.e. the gene trees matching the species tree, ‘phylogenetic topology’) was constructed using the total evidence nucleotide species tree (TENT) phylogeny, published by Jarvis et al. (2014). For species not included in the TENT phylogeny we used timetree (Hedges et al. 2015) divergence times to position these species in our phylogeny. The divergence constrained by migration scenario (‘migratory phenotype topology’) was constructed by clustering each phenotype (once exclusively focusing on migratory versus resident species for the restricted dataset; and also for the full dataset including other movement as a third phenotype category) in one separate branch while keeping the evolutionary relationships of the phylogenetic topology within each branch. Random divergence (‘random tree’) was generated shuffling branches randomly from the gene trees obtained, in order to avoid bias regarding the method of random trees generation by TOPD/fmts that only randomises taxa, but not branches for the statistical comparison. Restricting these analyses to exclusively Passerine species allowed us to analyse the effects of the evolutionary patterns of each candidate genes on a smaller scale. An example of the topologies is illustrated for the candidate gene *PER3* in Fig. S1.

Comparisons of these three focal topologies were carried out in TOPD/fmtS (Puigbò et al. 2007) using three different approaches: nodal, splits and disagree from the program. In brief, the ‘nodal approach’ counts the number of nodes that separate two taxa in a given topology and calculates the root mean squared deviation (RSMD) between each pair of trees. For identical topologies RMSD results in a value of zero. To calculate the significance of the RMSD obtained, TOPD/fmts calculates the distance between two contrasted tree pairs and 100 random trees obtaining one standard deviation (SD) confidence interval (CI). Compared topologies are characterised as statistically similar, within noise or different, depending on their distance with respect to CI. Specifically distances below CI denote similar topologies; distances above CI indicate statistical difference (distances around CI are within noise). The ‘disagree method’ characterises how many taxa need to be removed from the compared topology in order to end up with the exact same topologies for both trees (assessed as count of taxa/total taxa). Consequently, a value of 0/total indicates identical topologies. The ‘splits method’ evaluates if there are common branches between both trees, the lower the distance the more branches the tree pair shares.

#### d*N*/d*S* analysis

Accounting for the fact that different species might have found different ways to alter similar phenotypes in the same gene (i.e. different changes in sequence), we also analysed synonymous and non-synonymous mutations of all candidate genes across species. In order to pick up on putative selective pressures on candidate genes for migration, a gene-wide d*N*/d*S* analysis was carried out on the Datamonkey server (Pond and Frost 2005). We used three different datasets for each candidate gene: one including all the species, one restricted to migratory, and another restricted to non-migratory species.

Gene-wide d*N*/d*S* ratios (*w*) were estimated by maximum likelihood (ML) methods using a different model for each gene. Each model was obtained from the CMS module of the server. Neighbour Joining (NJ) phylogenies obtained for each candidate gene were used as input to assess likelihood of the tree comparing the neutral null model M1 (*w* < 1) and the model M2 that allows *w* > 1. Positive selection was assessed if the likelihood shows a *p* < 0.05.

To evaluate if lineages with migratory species show a signature of selection, a branch-specific analysis of dN/dS was also carried on Datamonkey with the Branch-Site REL program (Pond et al. 2011). The dataset for each candidate included migratory and non-migratory species. Branches under episodic diversifying selection were identified with a Holm–Bonferroni corrected *p* ≤ 0.05.

#### Structural features and predictors of migration

We used a linear regression analysis to test for a correlation between the genotype of migratory species at each focal candidate gene and both breeding latitude and migratory distance. Models for both predictors were run separately. The genotype used for each gene was the microsatellite length (as number of bases) of the 3′UTR of *ADCYAP1* and *CREB1*, or poly-Q (as number of predicted glutamine amino acids) on exon 20 of *CLOCK* and *NPAS*. For the *CLOCK* gene we included two separate polymorphic regions with variable poly-Q repeats in our analysis (both variable regions are located in the same exon). The significance of the fit was assessed with a simple linear regression, using a significance threshold *p* ≤ 0.05.

#### Within and across population variability in candidate gene sequence

Our comparative analyses focus exclusively on the sequence of one reference genome; inter-individual variation is not taken into account, mostly due to the limitation of available data to examine this level of variation. In order to make an attempt to see if variance within on candidate gene could be higher/lower in a specific migratory phenotype, we focused on *CLOCK* gene polymorphism, the only candidate gene with a sufficiently high number of individual sequence data available for several species (*n* = 10), including both migratory (*n* = 8) and resident (*n* = 2) species. Here we compare datasets of individually genotyped migratory species: flycatcher *Ficedula hypoleuca* (Saino et al. 2015; *n* = 226), willow warbler *Phylloscopus trochilus* ssp (unpublished data, *n* = 384), chiffchaff *Phylloscopus collybita* ssp (unpublished data, *n* = 56), nightingale *Luscinia megarhynchos* (Saino et al. 2015; *n* = 151), tree pipit *Anthus trivialis* (Saino et al. 2015; *n* = 144), barn swallow *Hirundo rustica* (Dor et al. 2011; *n* = 830), whinchat *Saxicola rubetra* (Saino et al. 2015, *n* = 374); and two non-migratory species: blue tit *Cyanistes caerulea* (Liedvogel et al. 2009; *n* = 950), great tit *Parus major* (Liedvogel and Sheldon 2010; *n* = 804). We compared averages and variances among different pairs of groups or species, employing a Welch *t* test and *F* test, respectively. We assume as statistically similar, distributions with a *p* > 0.001.

## Results

### Comparing phylogenetic, migratory phenotype and random topologies

**Fig. 1:**
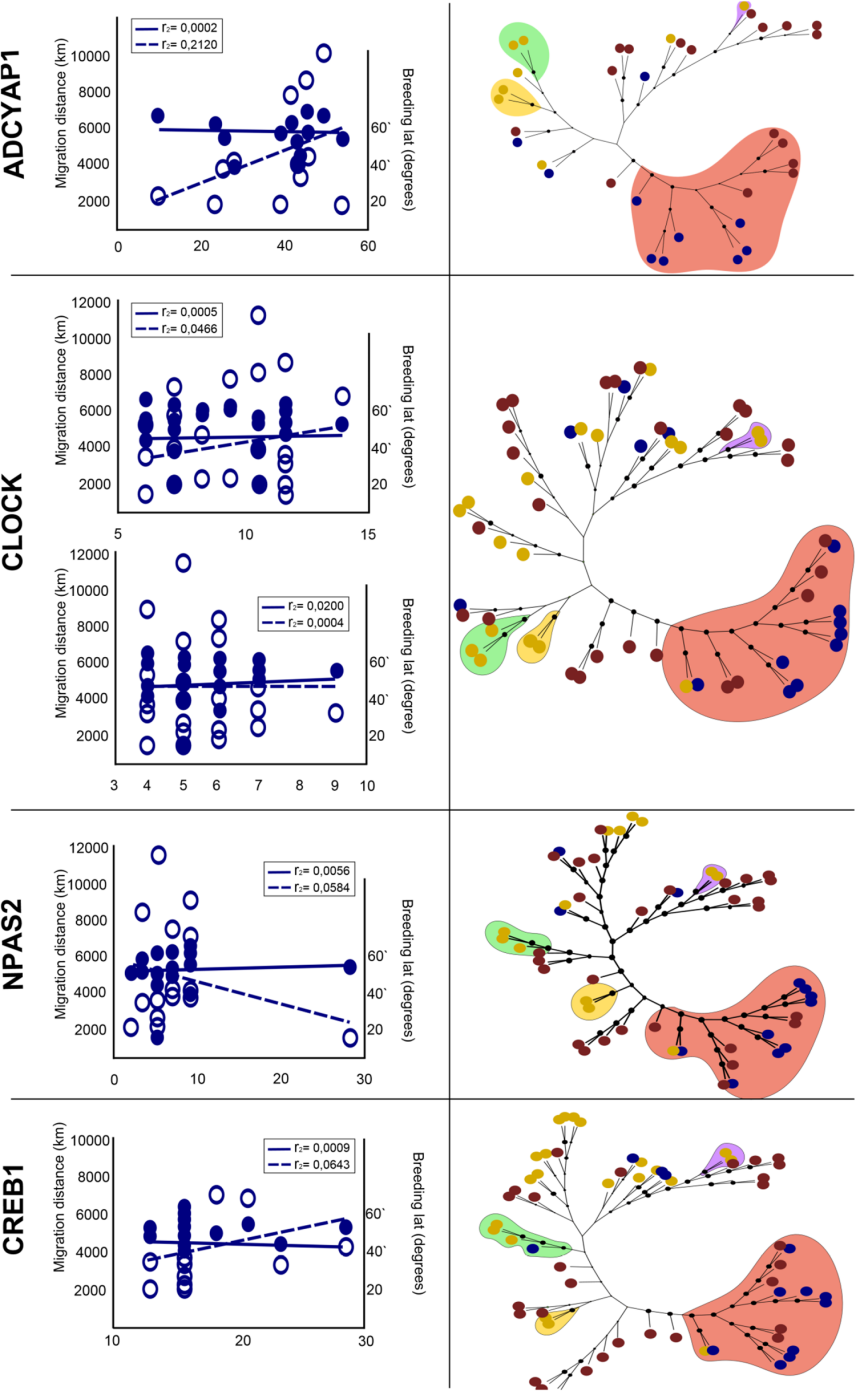
Clustering patterns of migratory candidate genes. *Right panel* shows gene phylogeny for each candidate gene. Neighbour Joining (NJ) analyses shown for the four most widely used candidate genes for migration. *Coloured dots* indicate migratory (*blue*), non-migratory (*red*) and partial migrant/dispersive (*yellow*) taxa: node support is indicated by the size of nodes. Coloured clouds highlighted in *red*, *yellow*, *green* and *purple* highlight represent *Passeriformes* (*red*), *Falconiformes* (*yellow*), *Accipitridae* (*green*) and *Anseriformes* (*purple*), respectively. *Left panel* shows repeat lengths of focal genetic variants of each candidate gene, exemplarily illustrated for the most widely used candidate genes in the context of migration: *ADCYAP1, CLOCK, NPAS* and *CREB1*. Genotype is plotted in relation to migratory distance (*open circles*, *dashed lines*) and breeding latitude (*filled circles*, *continuous lines*). *Dashed* and *continuous lines* indicate the trend for linear regression of repeat variation at the candidate locus versus migratory distance and breeding, respectively. Variation at the *CLOCK* genes is shown for both variable length repeat regions (also see Fig. S3). *R* squared values are state for fitted linear models

**Fig. 2:**
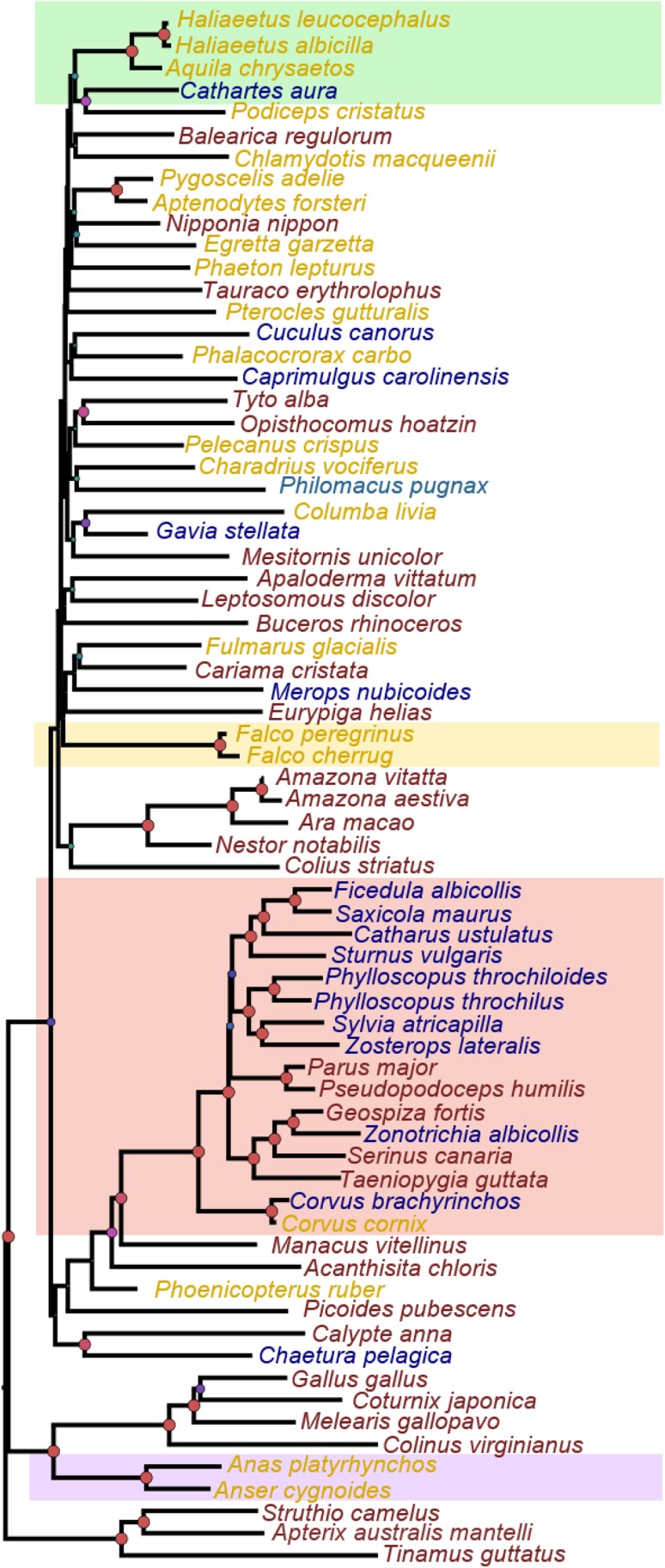
Phylogenetic tree of concatenated candidate gene sequences. Neighbour Joining topology of genomic sequences for all 25 candidate genes. *Colour scheme* and node support as in Fig. 1

We show constructed gene trees for a select number of candidate genes in Fig. 1, right column. In general, candidate gene trees do not separate migratory from non-migratory birds. Instead, they resemble the phylogenetic topologies expected based on Jarvis et al. (2014). Nonetheless, some groups of birds that comprised exclusively of migrants group together in most of the candidate gene trees. For example, *Falconifomes*, *Accipithridae* and some *Passeriformes* show a clustering pattern. Nevertheless, this is most likely due to their higher levels of similarity and common ancestry rather than relationships based on migratory phenotype (also see Fig. S2). Note that in addition to separate analyses for each of the 25 candidate genes, we also concatenated sequence data from all genes by species and constructed a combined candidate genes tree (Fig. 2). In this concatenated tree the clustering patterns persist in the aforementioned lineages.

**Fig. 3:**
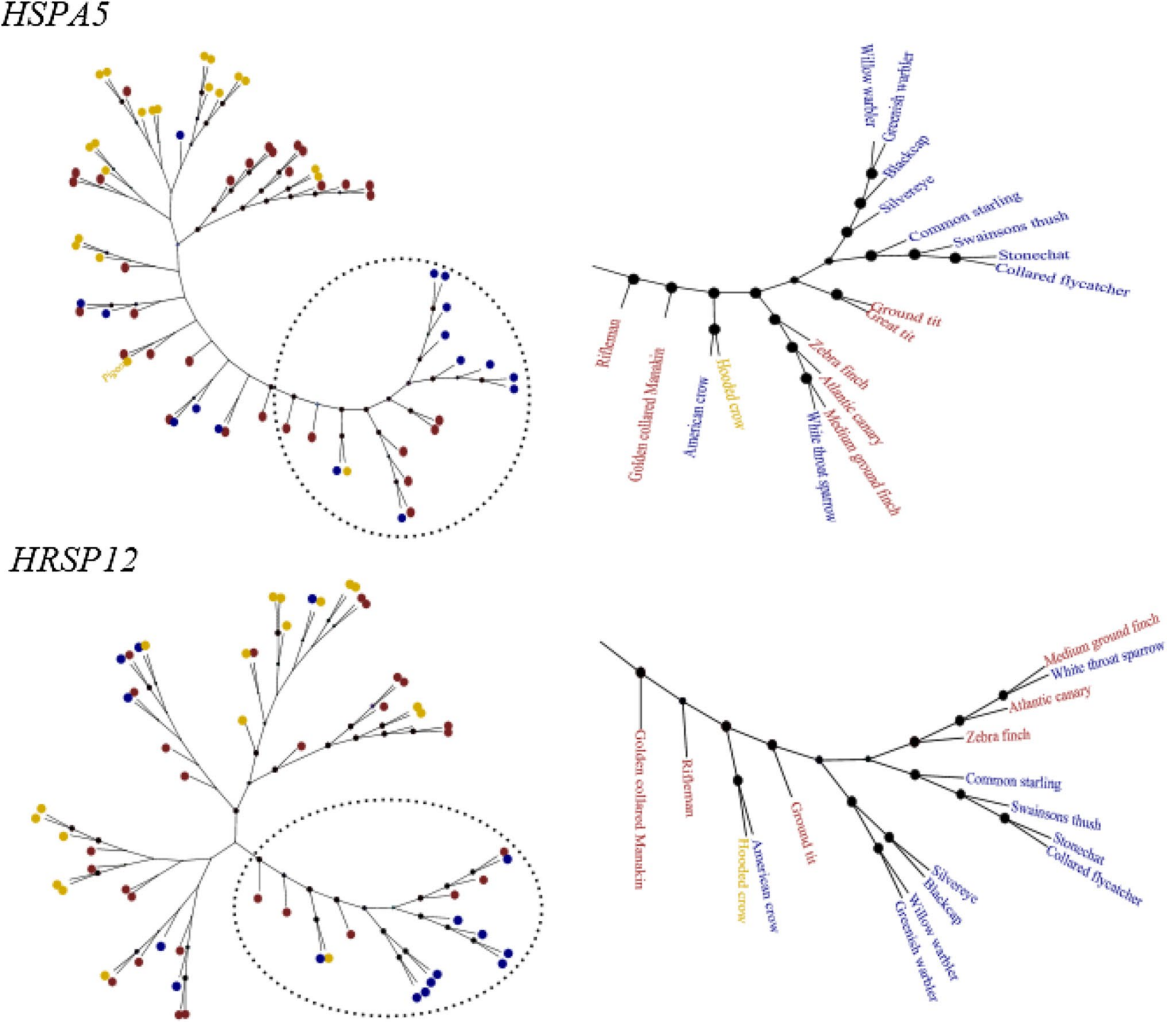
Topologies on a constrained phylogenetic scale. Clade-specific analysis exclusively focusing on all species within the genus Passerines. The candidate gene trees obtained from the Neighbour Joining analysis for HRSP12 and HSPA5, two candidate genes that follow clustering patterns consistently more similar to the migration topology than the speciation topology. *Colouring scheme* and node support as in Fig. 1

Results from nodal, splits and disagree methods for statistically distinguishing between phylogenetic, migratory phenotype and random trees can be found in Table 1 (full dataset). Our results clearly show that the phylogenetic topology tree provides the best fit to most gene trees. The lack of support for the migratory phenotype topology shows no evidence for a monophyletic origin for a migratory phenotype across avian taxa. This pattern did not change when restricting our analysis to only the extreme phenotypes, i.e. exclusively obligate migrants and clear non-migrants. We further zoomed into just one clade (specifically Passerines). Although overall, we see general patterns either more similar to the speciation phylogeny or not showing differences from a comparison with random topologies (see nodal approach in Table 1), the monophyletic clade analysis on Passeriformes indicates that respective gene topologies for some candidates, specifically *HRSP12* and *HSPA5,* are consistently more similar to the migration topology than the speciation topology (Fig. 3). This clustering pattern is also evident in the tree topologies (see Fig. S2) where most of the migrant species within Passerine tend to cluster in one branch. The other approaches did not show evidence for a general trend towards one or a set of candidate genes being recurrently involved in distinguishing migrants from non-migrants.

**Table 1:** Topology comparison among candidate gene trees and target trees

	RANDOM			SPECIATION			MIGRATION		
	Dis	Nod	Split	Dis	Nod	Split	Dis	Nod	Split
PASSERINES restricted dataset									
ADCYAP1	12/12	2.741378	1	1/12	**0.696311**	**0.111111**	2/12	0.912871	0.222222
CLOCK	12/12	2.383656	1	1/13	**1.450022**	**0.4**	1/13	1.450022	0.4
CRY1	8/15	2.146980	0.916667	1/15	**1.751190**	**0.416667**	1/15	1.751190	0.416667
CRY2	17/17	3.226727	1	4/17	2.711631	**0.5**	4/17	**2.637401**	0.5
DRD4	17/17	3.391165	0	0/17	**0**	**0**	5/17	2.730546	0.5
HRSP12	15/15	2.700970	1	3/15	1.825742	0.5	2/15	**1.621287**	**0.416667**
HSP90B1	14/14	2.896522	1	1/14	**1.657386**	**0.364656**	3/14	1.675089	0.374074
HSPA5	16/16	3.345395	1	6/16	2.534758	**0.4**	3/16	**1.897367**	0.4
HSPA8	14/14	3.275938	1	1/14	**2.305879**	**0.435897**	3/14	2.399986	0.564103
NEK2	14/15	2.640600	0.638889	1/15	**1.067071**	**0.272222**	3/15	1.977253	0.438889
NFIL3	15/16	3.221151	1	2/16	**2.416335**	**0.5**	4/16	2.507021	0.5
PARL	17/17	3.429972	1	5/17	2.667892	0.642857	4/17	**2.546624**	**0.5**
PER2	13/13	2.480695	1	1/14	**1.500915**	**0.363636**	1/14	1.500915	0.363636
PER3	17/17	3.564531	1	5/17	2.825826	0.642857	4/17	**2.662374**	**0.5**
SLC2A1	12/12	2.424005	1	2/12	**1.299174**	**0.309259**	5/12	1.371361	0.546296
AANAT	16/16	2.966479	1	3/16	**2.081666**	**0.307692**	4/16	2.677063	0.692308
CSNK1E	13/13	3.125577	1	4/13	2.124340	**0.5**	4/13	**1.860521**	0.5
SLC1A3	12/14	3.119223	1	1/14	**2.401755**	**0.465397**	1/14	2.568437	0.472222
HSP90AA1	13/15	3.072418	0.823900	3/15	**1.345224**	**0.5**	5/15	2.042943	0.35
TTR	14/15	3.034009	1	2/15	**2.113851**	**0.407280**	2/15	2.193276	0.437451
YPEL1	13/13	2.916833	0.990741	3/13	**1.874818**	**0.5**	3/13	1.894358	0.5
NPAS2	10/10	2.653709	1	2/10	**1.341210**	**0.322046**	4/10	1.674568	0.386420
ARNTL	12/12	3.286592	1	2/12	**2.412694**	**0.4**	2/12	2.664591	0.4
CPNE4	13/14	3.221151	1	2/14	**2.416335**	**0.527778**	2/14	2.707021	0.472222
CREB1	17/17	3.735757	1	4/17	**2.711631**	**0.5**	4/17	2.902374	0.5
MIGRANTS versus NON-MIGRANTS restricted dataset									
ADCYAP1	26/26	3.478727	0.9565	5/27	**2.951652**	**0.375**	9/27	3.148735	0.5
CLOCK	40/40	5.640058	1	11/41	**5.180639**	**0.578947**	17/41	5.264237	0.631579
CRY1	39/39	5.528725	0.9722	7/40	**5.137968**	**0.621622**	17/40	5.142956	0.648649
CRY2	41/41	5.931387	0.9766	10/41	5.340413	0.683058	17/41	**5.126161**	**0.618569**
DRD4	41/41	5.086807	1	3/17	**0.469668**	**0.071429**	12/42	4.301095	0.512821
HRSP12	42/42	4.994887	0.9744	11/43	5.041138	**0.675**	23/43	**4.723354**	0.75
HSP90B1	46/47	5.639855	0.9852	16/47	**5.230941**	**0.640355**	21/47	5.391719	0.666943
HSPA5	47/47	5.483471	1	12/48	**5.055016**	**0.622222**	27/48	5.831560	0.733333
HSPA8	44/44	5.468022	1	13/44	**4.792124**	**0.670420**	18/44	4.877529	0.713964
NEK2	44/44	4.559393	0.9783	16/44	**4.066146**	**0.476974**	20/44	4.206486	0.565789
NFIL3	46/47	5.878573	0.9852	15/47	**5.185939**	**0.661762**	21/47	5.276429	0.686346
PARL	42/42	4.882503	0.9762	17/42	**4.423420**	**0.525190**	17/42	4.518643	0.593409
PER2	42/42	5.092866	1	13/43	**4.831376**	**0.65**	22/43	5.369616	0.675
PER3	46/46	4.579834	1	23/46	4.538616	0.697674	15/46	**3.413103**	**0.558140**
SLC2A1	28/28	5.259547	0.9853	13/28	**5.003874**	**0.646401**	16/28	5.037441	0.693649
AANAT	38/38	4.836443	1	10/39	**4.577714**	**0.611111**	20/39	5.174160	0.833333
CSNK1E	40/40	6.099601	1	20/40	5.006534	0.729730	13/40	**4.580897**	**0.594595**
SLC1A3	46/46	5.205493	0.9822	13/46	**5.003494**	**0.648874**	26/46	5.078642	0.691216
HSP90AA1	39/39	4.910991	0.9556	19/39	**4.893480**	**0.573299**	26/39	4.915253	0.631431
TTR	21/22	5.185258	0.9915	12/22	**4.888213**	**0.665140**	19/22	4.990166	0.712988
YPEL1	47/48	4.874233	0.9928	13/48	**4.553259**	**0.395**	22/48	4.683716	0.619087
NPAS2	40/40	5.479820	1	19/40	**4.936480**	**0.660727**	21/40	5.174525	0.678437
ARNTL	36/36	4.673910	0.9848	17/36	**4.342727**	**0.566613**	28/36	4.646077	0.572446
CPNE4	38/39	5.878573	0.9852	18/39	**5.185939**	**0.661762**	25/39	5.276429	0.676346
CREB1	40/48	6.699674	1	24/48	**5.973568**	0.644444	15/48	6.074231	**0.511111**
Full dataset									
ADCYAP1	30/34	3.794169	0.935484	8/35	3.906039	**0.468750**	15/35	**3.467496**	0.593750
CLOCK	59/59	5.931323	1	20/60	**5.510288**	**0.684211**	27/60	5.647208	0.701754
CRY1	56/60	5.894122	0.982456	23/62	**5.500493**	**0.677966**	28/62	5.589141	0.677966
CRY2	61/61	5.690368	0.991729	19/61	5.419998	0.676801	24/61	**5.307191**	**0.662440**
DRD4	60/60	5.837537	1	3/17	**0.469668**	**0.071429**	20/61	4.578520	0.568966
HRSP12	62/62	5.237258	1	27/63	5.349055	**0.7**	30/63	**5.082625**	0.733333
HSP90B1	67/67	4.944896	0.965674	25/67	**4.556093**	**0.604968**	30/67	4.976348	0.672276
HSPA5	67/67	6.240832	1	18/68	**5.691938**	**0.630769**	35/68	5.965220	0.692308
HSPA8	62/63	5.620202	0.985844	22/63	**5.125876**	**0.667334**	27/63	5.353811	0.682397
NEK2	65/66	6.254055	0.983173	25/66	**5.649675**	**0.651162**	27/66	5.737866	0.607372
NFIL3	67/67	5.970355	0.991207	21/67	**5.528167**	**0.637798**	32/67	5.724862	0.624456
PARL	66/66	5.523274	0.989792	20/66	**5.203310**	**0.632417**	29/66	5.403632	0.628094
PER2	59/59	5.391185	1	22/60	**5.182543**	**0.666667**	28/60	5.781717	0.684211
PER3	66/66	5.478672	1	30/67	5.203242	0.671875	20/67	**4.601044**	**0.562500**
SLC2A1	41/41	5.860697	0.987179	15/41	**5.275366**	**0.663248**	20/41	5.524032	0.670378
AANAT	54/54	5.090447	1	14/55	**4.810622**	**0.653846**	30/55	5.136389	0.788462
CSNK1E	51/55	5.950073	0.961538	25/55	4.951618	0.692308	19/55	**4.825159**	**0.634615**
SLC1A3	65/65	4.862746	0.967742	16/65	**4.708164**	**0.576480**	25/65	4.557352	0.647752
HSP90AA1	52/53	5.206538	0.972647	17/53	**4.972273**	**0.610309**	23/53	4.991282	0.657823
TTR	22/22	6.378516	0.965144	11/22	**5.565637**	**0.666466**	14/22	5.380218	0.582933
YPEL1	48/48	5.687568	0.994152	16/48	**5.453279**	**0.687392**	30/48	5.639658	0.704351
NPAS2	56/56	5.182652	0.980130	20/56	**4.961226**	**0.619555**	31/56	4.986559	0.622692
ARNTL	55/56	5.949312	0.974225	17/56	**5.253574**	**0.667268**	24/56	5.519978	0.648638
CPNE4	54/54	5.503541	0.991195	19/54	**5.202493**	**0.676264**	27/54	5.373337	0.696865
CREB1	61/67	6.806959	0.968750	29/67	6.179656	0.640625	18/67	**5.935276**	**0.531250**

To contrast topology patterns we compared single gene trees derived for each candidate gene with three target trees: (1) randomly generated trees based on randomly permutating braches of single gene tree topology ‘random topology’; (2) genes based on overall phylogeny (Jarvis et al. 2014), thus representing evolutionary divergence among all the species included in the study ‘phylogenetic topology’, and (3) topology classified by migratory behaviour, i.e. an artificial tree clustering migratory species and non-migratory species in two different branches ‘topology based on migratory phenotype’

*Bold* characters highlight the most similar topology for each candidate gene tree for the three methods. Tables shown for three datasets: Monophyletic analysis on Passerines; exclusively contrasting obligate migrants versus clear residents; and the full dataset including the third phenotype category with partial migrants, dispersive species

Topologies were compared and analysed by three methods included in the TOPD software: *Dis* disagree, *Nod* nodal, *split* split (see “Materials and methods” for details)

* and ** Denote significance below and above one SD of 100 randomly generated trees, respectively

Recall that we also concatenated sequence data from all genes by species and constructed a complete gene tree. This concatenated phylogeny shows that most of the taxonomic groups clustering migratory species together are statistically well supported and show larger branch lengths. This could suggest an evolutionary process of acceleration in these lineages for some of the candidate genes.

### Selection in candidate genes is not related to migration

To identify selective forces in lineages with migratory species, we performed a gene-wide and branch-specific d*N*/d*S* analysis. The gene-wide analysis for selection in candidate genes across all bird species indicates that *CLOCK, DRD4, NEK2, HSPA5* and *CSNKE1*, have been under positive selection at *p* < 0.05 (Table 2). Nonetheless, and important within the focus of our analysis, this signature does not seem to be linked to the migratory phenotype in any of our comparisons, as independent datasets of separately analysed migratory, partial migratory and non-migratory species generally do not show any signature of selection. The only exception here was the *CLOCK* gene that showed significant values for selection in partial migrants. Thus, despite the general overall pattern of selection across all avian clade, none of these candidate genes showed lineages under selective pressure.

**Table 2:** Gene-wide dN/dS analysis of migratory gene candidates

Gene	Phenotype	lnL M1	lnL M2	LRT	*P* value
ARNTL	ALL	−12014.300	−12014.300	1.30E−04	1.000
	Migratory	−2443.540	−2443.540	8.43E−05	1.000
	Partial migrants	−5751.000	−5883.250	3.42E−03	1.000
	Residents	−9022.375	−9022.055	1.17E+00	0.623
CLOCK	**ALL**	−**17203.100**	−**17199.300**	**7.49E+00**	0.0236713**
	Migratory	−2784.523	−2784.444	2.89E−04	0.899
	**Partial migrants**	−**6084.900**	−**6801.840**	**6.13E+00**	0.0467**
	Residents	−12321.600	−12321.600	1.27E−04	1.000
PER3	ALL	−41720.300	−41718.500	3.49E+00	0.175
	Migratory	−2124.250	−2124.250	2.34E−06	1.000
	Partial migrants	−5883.250	−5883.250	6.48E−05	1.000
	Residents	−13206.766	−13206.727	7.11E−02	0.969
CRY1	ALL	−11297.000	−11297.000	6.54E−05	1.000
	Migratory	−2618.571	−2618.509	5.14E−01	0.949
	Partial migrants	−6543.055	−6543.055	2.94E−05	0.999
	Residents	−7465.354	−7465.354	4.34E−05	1.000
CRY2	ALL	−12140.600	−12140.600	−5.26E−04	1.000
	Migratory	−2718.098	−2718.030	2.57E−01	0.869
	**Partial migrants**	−**5137.220**	−**5134.740**	**4.96E+00**	**0.084**
	Residents	−11345.504	−11345.444	1.11E−01	0.952
NEK2	**ALL**	−**15013.300**	−**15008.900**	**8.86E+00**	0.0119051**
	Migratory	−3123.490	−3123.400	1.03E+00	0.798
	Partial migrants	−6882.490	−6882.400	2.48E−06	1.000
	Residents	−13729.370	−13729.286	1.55E−01	0.932
NPAS2	ALL	−9773.415	−9773.415	3.58E−07	1.000
	Migratory	−2788.055	−2787.988	6.78E−04	1.000
	Partial migrants	−6711.240	−6711.240	5.43E−06	1.000
	Residents	−8384.493	−8384.493	4.83E−05	0.998
PER2	ALL	−34024.900	−34024.900	2.68E−04	1.000
	Migratory	−2703.313	−2703.248	3.57E−04	0.975
	Partial migrants	−5601.859	−6548.185	−1.29E−04	1.000
	Residents	−14439.024	−14438.968	1.02E−01	0.955
DRD4	**ALL**	−**9382.370**	−**9377.070**	**1.06E+01**	0.00499358**
	**Migratory**	−**3260.480**	−**3257.910**	**5.14E+00**	**0.077**
	Partial migrants	−5392.430	−6882.400	5.45E−06	1.000
	Residents	−6979.373	−6979.033	1.25E+00	0.599
AANAT	ALL	−5698.000	−5698.000	2.84E−05	1.000
	Migratory	−2699.671	−2699.604	6.46E−02	0.914
	Partial migrants	−6545.730	−6545.730	2.02E−05	0.998
	Residents	−4721.560	−4721.560	−7.37E−05	1.000
CPNE4	ALL	−11228.606	−11228.588	3.60E−02	1.000
	Migratory	−2452.620	−2452.575	5.76E−04	1.000
	Partial migrants	−5633.000	−6539.900	3.20E−04	0.999
	Residents	−9385.890	−9385.890	3.73E−04	0.999
HSPA5	**ALL**	**−16331.700**	**−16328.200**	**6.92E+00**	0.0314173**
	Migratory	−2707.037	−2706.969	3.26E−02	0.796
	Partial migrants	−5595.625	−6566.695	1.26E−03	0.993
	Residents	−12404.581	−12404.509	1.33E−01	0.942
HSPA8	ALL	−11781.944	−11781.943	2.92E−03	1.000
	Migratory	−2703.351	−2703.283	1.29E−01	0.866
	Partial migrants	−5594.500	−6547.055	−5.42E−05	1.000
	Residents	−7568.080	−7568.080	8.25E−05	1.000
HSP90B1	ALL	−16126.900	−16126.900	−9.01E−05	1.000
	Migratory	−2780.990	−2780.900	4.24E−04	1.000
	Partial migrants	−5603.844	−6540.379	2.81E−04	0.998
	Residents	−15017.600	−15017.300	5.54E−01	0.758
HSP90AA1	ALL	−14902.200	−14902.200	2.91E−05	1.000
	Migratory	−13307.269	−13307.209	1.11E−01	0.951
	Partial migrants	−5596.750	−6586.335	2.21E−03	0.990
	Residents	−13025.049	−13024.987	1.14E−01	0.950
SLC2a1	ALL	−3747.905	−3747.905	−1.05E−03	1.000
	Migratory	−2703.354	−2703.286	1.93E−01	0.832
	Partial migrants	−6535.469	−6535.469	2.20E−05	0.977
	Residents	−2483.450	−2483.450	3.48E−04	0.954
SLC1a3	ALL	−9229.518	−9229.518	2.19E−06	1.000
	Migratory	−2681.245	−2681.178	5.17E−04	0.943
	Partial migrants	−6547.055	−6547.055	−2.15E−04	1.000
	Residents	−5723.150	−5722.510	2.34E+00	0.246
PARL	ALL	−5321.898	−5321.143	1.51E+00	0.621
	Migratory	−2695.975	−2695.908	2.57E−01	0.859
	Partial migrants	−5602.781	−6543.055	1.18E−03	0.999
	Residents	−3519.020	−3519.020	−4.90E−06	1.000
TTR	ALL	−10543.983	−10543.914	1.38E−01	0.999
	Migratory	−2699.665	−2699.597	1.45E−01	0.873
	Partial migrants	−5611.500	−6504.243	−2.44E−05	1.000
	Residents	−7637.370	−7637.370	2.34E−04	0.989
YPEL1	ALL	−9350.850	−9350.062	1.58E+00	0.532
	Migratory	−2692.279	−2692.213	1.29E−01	0.733
	Partial migrants	−6545.289	−6545.289	8.76E−06	0.989
	Residents	−6270.698	−6270.538	5.86E−01	0.811
HRSP12	ALL	−6547.120	−6547.110	−2.40E−03	1.000
	Migratory	−2745.243	−2745.243	3.24E−06	0.987
	Partial migrants	−5604.125	−6545.289	8.76E−06	0.989
	Residents	−4936.372	−4936.012	1.32E+00	0.576
CREB1	ALL	−5330.580	−5330.580	6.44E−05	1.000
	Migratory	−2786.289	−2786.289	6.08E−05	0.999
	Partial migrants	−6504.243	−6504.243	−1.06E−04	1.000
	Residents	−3287.980	−3287.980	7.82E−04	1.000
CSNKE1	**ALL**	**−5495.620**	**−5490.040**	**1.12E+01**	0.00376182**
	Migratory	−2784.055	−2784.055	1.80E−04	0.921
	Partial migrants	−6546.958	−6546.958	−4.89E−04	1.000
	Residents	−4149.594	−4149.514	2.93E−01	0.906
ADCYAP1	ALL	−1903.400	−1903.540	−2.82E−01	1.000
	Migratory	−2781.379	−2781.379	2.14E−05	0.859
	Partial migrants	−6555.992	−6555.992	5.65E−06	1.000
	Residents	−2028.490	−2028.490	3.43E−05	1.000
NFIL3	ALL	−9638.910	−9638.910	1.55E−04	1.000
	Migratory	−2788.055	−2788.055	5.14E−01	0.949
	Partial migrants	−5565.000	−6711.240	−2.83E−05	1.000
	Residents	−8583.364	−8583.304	1.11E−01	0.952

Significance of positive selection was assessed with *p* < 0.1 indicated with bold font and *p* < 0.05 indicated with **

### Association between structural features and predictors of migration

Alignments of the genomic sequences (Fig. S3) of any of the 25 gene candidates did not separate migratory and non-migratory species. *ADCYAP1* has variable sequence lengths of a microsatellite with an AG repeat at the 3′ UTR region ranging from 26 to 56 bp for migratory species, and 10–54 bp for non-migratory species. *CREB1* also has a microsatellite with a TG/CG repeat motive at the 3′ UTR region, ranging from 12 to 26 bp in migratory and 14–42 bp in non-migratory species, respectively (the longest length variant was exclusively found in tits). *NPAS* has one variable region of poly-Q repeats, in migratory species; this has length variation between 6 and 10 amino acids (aa), in non-migratory species the repeat length varied between 5 and 12 aa repeats. The *CLOCK* gene has 2 poly-Q variable regions, both located on exon 20. The first region (R1) shows length variation between 6 and 13 poly-Q repeats in migrants and 4 to 14 repeats in non-migratory species. Length polymorphism in variable region two (R2) varied between 4 and 9 poly-Q repeats in both phenotypes. Lengths variation in neither of the focal candidates showed significant differences between migratory and non-migratory birds.

The intra-specific analysis focusing on individual variability of *CLOCK* gene polymorphism shows a tendency for higher variability in migratory species (Fig. S4); however, given that only two non-migratory species were included (namely great tits and blue tits—great tits being mostly monomorphic, blue tits highly variable), this does not allow us to draw any statistically supported conclusions (Table S3). Nonetheless, this individual based analysis of variability suggests that most common allele lengths (i.e. number of poly-Q repeats) varied considerably between species, as did the overall degree of variability within species (Fig. S4, Table S3). For example, migratory species like *H. rustica* and *F. hypoleuca*, show constrained levels of variability, being mostly monomorphic, while *C. caeruleus*, a non-migrant species, shows a degree of variance comparable to those migrant species. Statistical differences were found between the group of migrant and non-migrant species. Nonetheless when the comparisons were performed between individual species, migrant species and non-migrant species showed statistical differences, but also comparisons between a migrant and a non-migrant species did not show statistical differences (see Table S3).

We used linear models to quantify the correlation of structural repeats (poly-Q in *CLOCK* and *NPAS2*; and allele lengths in *ADCYAP1* and *CREB1*) and both breeding latitude and migratory distance (Fig. 1, left column). The fit of the data to a linear regression model did not show significant associations between genotype and migratory phenotype (Table 3). The best fit, albeit not significant, that was following a similar pattern as earlier work showing a correlation between candidate gene variation and migratory phenotype results from our comparison between *ADCYAP1* genotype variation in relation to migratory distance (*r* ^2^ = 0.2120, *p* = 0.01132). Although not significant, here the regression trend is in line with earlier studies, and might be taken as support for earlier findings reporting genotype variation at this locus with relevance to the migratory phenotype on the within-species level.

**Table 3:** Predictors of linear models

	*df*	Res err	*F*	*p*
ADCYAP1 vs distance	11	2228	2.962	0.1132
ADCYAP1 vs breeding	11	7.299	0.05144	0.8252
CREB1 vs distance	13	2093	0.8929	0.3619
CREB1 vs breeding	13	6.126	0.2411	0.6316
CLOCK R1 vs distance	23	2675	1.125	0.3
CLOCK R1 vs breeding	23	12.8	0.01369	0.9079
CLOCK R2 vs distance	21	2726	0.3082	0.5846
CLOCK R2 vs breeding	21	17.73	0.1876	0.6693
NPAS2 vs distance	17	2701	0.647	0.432
NPAS2 vs breeding	17	11.29	0.0788	0.7825

For every comparison *df* degree of freedom, *res* residual error, *F* statistic and significance levels are shown

Significance at *p* < 0.05

## Discussion

We used publicly available archived genomic data from non-migratory and migratory bird species to test for the presence or absence of a general clustering pattern in candidate genes for migration. In a cross-species comparative approach, we characterised sequence features in 25 candidate genes in a dataset including birds across all orders of Aves. Our study thus illustrates the potential of public genomic data to test for general patterns of migratory gene candidates in a cross-species comparative context. Despite this powerful dataset we were not able to identify genetic variation that allowed us to distinguish migratory from non-migratory birds based on sequence difference in any of the candidate genes included here.

Most patterns we recover based on candidate gene sequence variation were driven by species phylogeny, and did not show indications separating migratory and non-migratory birds. This could suggest that candidate genes do not play a general role in controlling migration across the full avian clade, or that patterns cannot be evident in a wide phylogenetic analysis. In addition to analysing each candidate gene individually, we also analysed concatenated sequence data from all candidate genes, which also did not allow clear separation of migratory and non-migratory birds. The latter finding might not be too surprising, as this scenario would assume divergence of candidate genes among lineages in parallel to species divergence, a rather unlikely scenario.

In order to address the fact that we cannot exclude that the relevance and contribution of certain traits may differ between avian lineages in a way that different traits may be controlled by different mutations in the same genes, and/or varying patterns of epistatic interactions with other genes, we also screened for putative features of selection within these candidate genes. However, our dN/dS approach did not allow identifying patterns of selection at either the gene-wide or branch-sites level for the migratory lineages. This result does not provide support for lineage-specific selection on the gene candidates.

Indicative patterns might get lost if not approached at the appropriate phylogenetic scale. Our more focused analyses on a restricted data assembly exclusive to the Passeriformes allow aims to address this issue at least partly allowing us to potentially recover general trends towards some or all candidate genes being recurrently involved in the control of migration. Analyses limited to this family show improved resolution and come close to the margin of randomness in nodal comparisons. However, even on this more focal scale only a few of these comparisons show better values than the phylogeny species tree, suggesting that even on a more constrained phylogenetic scale, the genes keep following the trends of phylogeny or speciation rather than clustering according to migratory phenotype.

Most studies of candidate genes look for correlates of length polymorphism in a limited number of species. Our approach aims to extend this framework to assessing sequence variability in candidate genes to a broader evolutionary scale. However, our data do not uncover statistical differences between species with different phenotypes, neither do they recover correlations with specific migratory features, specifically distance and breeding latitude. *ADCYAP1* did show a tendency for positive, though non-significant, correlation with migratory distance in our dataset, supporting earlier findings in species-specific analyses. As the length polymorphism in this gene is located in a 3′ UTR this could suggest that a specific pattern of gene expression is playing a role for this gene, and this feature might be relevant in long-distance migrants.

Our focus is put on general sequence differences in candidate genes for migration across species, but we also note that there is a lot of variation in the level of individual and inter-population diversity across avian species in many of the candidate genes included here, such as *CLOCK* poly-Q. Within-species variability at polymorphic loci often is high in some, but certainly not all species, irrespective of migratory phenotype. Previous work has suggested that variability in lengths polymorphism of *CLOCK* related to the timing of migration is enhanced in migratory birds (Saino et al. 2015). Our analyses testing for a role of inter-individual variation in *CLOCK* lengths polymorphism between migratory and non-migratory species falls short in strengthening this suggestion. We detect similarly high levels of polymorphisms as well as scenarios with almost monomorphic genotypes for various species, irrespective of migratory phenotype. This could mean that the observed variability in *CLOCK* is more tightly linked to other parameters that shape the variability of the migratory phenotype, such as breeding latitudes, distances and timing regimes, rather than specifically controlling timing traits in a migration context.

But even when leaving inter-individual variability aside and focusing on just one reference sequence, we expect general patterns that allow separating migrants from non-migratory species of suggested candidate genes for migration to show up if they were in fact under parallel selection. Our results do not support this hypothesis and thus highlight limitations and question the usefulness of a candidate gene approach in the context of understanding migratory behaviour. Consequently, we call for caution and highlight the limitation of candidate gene approaches in macro-evolutionary contexts, as in most cases functionality cannot be easily inferred. Also, most candidate gene studies do not allow for a clear distinction from genetic drift, come with uncertainty about the amount of loci involved in the trait and do not allow controlling for possible effect of linkage disequilibrium. Our study further points out how this approach can erroneously simplify a highly complex phenotype that most probably is a multilocus trait.

One further point we feel is especially important to keep in mind when using a candidate gene approach in the context of migration: many candidate genes have been identified in genetic model organisms—none of that migrate or show any correlate to a migratory phenotype, and functional validation or relevance is mostly lacking. In many of these studies, the subset of candidate genes for migration were selected based on their assumed effect on anticipated candidate traits that feed into the complex phenomenon of migration. Importantly, one possibility explaining the lack of pattern might be the limitation of the starter set of ad hoc hypothesised candidate genes we investigate and may not be completely unexpected. Once more genomic data for migratory and non-migratory species will become available, the genomic toolbox allowing to investigate the genetic basis of migratory traits will grow and allow for an increasingly more informed list of de novo identified candidates to be tested in migratory birds.

## Electronic supplementary material

Below is the link to the electronic supplementary material.

Figure S1 Trees employed for topology comparison. Seven trees are generated for each candidate gene. Migratory taxa are highlighted in blue. Here the gene tree for *PER3* obtained from the Neighbour Joining Analysis (A) is compared to a migratory phenotype topology (B), phylogenetic topology (C), random (D). (E) Shows Topology comparisons exclusively based on the reduced dataset exclusively containing obligate migrants and non-migratory species (E), as well as the pattern for the full dataset including all three phenotype classes (F) (SVG 366 kb)

Figure S2 Simplified topologies of all gene candidates. The gene trees obtained from the Neighbour Joining analysis for each candidate gene. Colouring scheme and node support as Figure 1 (SVG 2235 kb)

Figure S3 Cross-species comparative pattern of length polymorphisms at candidate genes for migration exemplarily illustrated for the four most widely used candidates (*ADCYAP1, CLOCK, NPAS* and *CREB1*). Upper panel (highlighted by a blue bar to the left) shows migratory species aligned by decreasing number of repeats at the variable locus; lower panel comprises non-migratory species arranged by increasing length polymorphisms. Only one sequence per species was used as reference (SVG 319 kb)

Figure S4. Intra-specific variation of the *CLOCK* gene in migratory and non-migratory species. Boxplot of allele lengths at the poly-Q region in migratory (Barn swallow, Chiffchaff, Nightingale, Pied flycatcher, Tree pipit, Whinchats and Willow warblers) and non-migratory species (Blue tit, Great tit). Lines indicate the most common allele of each species (EPS 791 kb)

Table S1 Reference information for all avian species included in the cross-species comparison. NCBI ID reference is included if available. Breeding latitude is stated as degrees from equator. Migratory distance calculated as kilometres between breeding and wintering grounds (only for migratory species). Category is a classification into 0: completely non-migratory (resident), 1: partial migratory/dispersive, 2: obligate migratory species. Distance and breeding latitude obtained following an approach explained in Delmore et al. 2015b (XLS 42 kb)

Supplementary material 6 (DOCX 15 kb)
