# Supplementary material for: Candidate genes for migration do not distinguish migratory and non-migratory birds
Source: J Comp Physiol A Neuroethol Sens Neural Behav Physiol. 2017 Jun 5;203(6):383–97. doi: 10.1007/s00359-017-1184-6 (PMC5522501; doi:10.1007/s00359-017-1184-6)
Supplement: Supplementary file 6 — Supplementary material 6 (DOCX 15 kb) [file 359_2017_1184_MOESM6_ESM.docx]

**Table S2**. Number of bird species per candidate gene for which we were able to obtain full sequence information to be included in respective analyses.

| **Gene** | **Number of spp.** |
| --- | --- |
| AANAT | 55 |
| ADCYAP1 | 37 |
| ARNTL | 63 |
| CLOCK | 61 |
| CPNE4 | 60 |
| CREB1 | 67 |
| CRY1 | 64 |
| CRY2 | 61 |
| CSNK1E | 55 |
| DRD4 | 61 |
| HRSP12 | 63 |
| HSP90B1 | 68 |
| HSPA5 | 68 |
| HSPA8 | 63 |
| HSPA90AA1 | 63 |
| NEK2 | 66 |
| NFIL3 | 68 |
| NPAS | 58 |
| PARL | 66 |
| PER2 | 60 |
| PER3 | 67 |
| SLC1A3 | 53 |
| SLC2A1 | 41 |
| TTR | 22 |
| YPEL1 | 48 |

**Table S3. Welch t test and F-test for intra- and inter specific comparison on the polymorphic *CLOCK* locus.** Comparisons of *CLOCK* gene variability among and between migratory and non-migratory species.

|  | **t-test** | | | **F-test** | | | |
| --- | --- | --- | --- | --- | --- | --- | --- |
| **Comparison** | **t value** | **df** | **p** | **F value** | **df num** | **df den** | **p** |
| **Migratory** *vs* **non migratory** | -69,779 | 4733,3 | <0.0001 | 6,1509 | 3579 | 3503 | <0.0001 |
| **Great tit** *vs* **Blue tit** | -86,2 | 2646,3 | <0.0001 | 5,5388 | 1895 | 1607 | <0.0001 |
| **WW** *vs* **Nightingale** | 7,5993 | 475,1 | <0.0001 | 1,4549 | 301 | 753 | <0.0001 |
| **Flycatcher** *vs* **Bluetit** | -8,0875 | 834,3 | <0.0001 | 0,62122 | 451 | 1895 | <0.0001 |
| **Fly** *vs* **Great** | -65,692 | 526,71 | <0.0001 | 3,4408 | 451 | 1607 | <0.0001 |
| **WW** *vs* **Chiff abi** | -2,7165 | 69,38 | 0,008323 | 1,2333 | 61 | 753 | 0,2303 |
| **WW** *vs* **Chiff tri** | 0,84535 | 54,38 | 0,4016 | 1,2378 | 49 | 753 | 0.2646 |
| **Chiff tri** *vs* **Night** | -4,2575 | 69,497 | <0.0001 | 0,85082 | 49 | 301 | 0,5007 |
| **Chiff abi** *vs* **Night** | -6,3066 | 93,018 | <0.0001 | 0,8477 | 61 | 301 | 0,4404 |
| **Night** *vs* **Blue** | -20,158 | 402,62 | <0.0001 | 1,0026 | 301 | 1865 | 0,9597 |
| **Great tit** *vs* **Whinchat** | 1,813 | 476,43 | 0,07046 | 0,27898 | 1607 | 415 | <0.0001 |
| **Chiff abi** *vs* **Chiff tri** | -1,2265 | 104,95 | 0,2227 | 0,99633 | 61 | 49 | 0,9813 |
